# Cannabinoid Receptors Overexpression in a Rat Model of Irritable Bowel Syndrome (IBS) after Treatment with a Ketogenic Diet

**DOI:** 10.3390/ijms22062880

**Published:** 2021-03-12

**Authors:** Isabella Gigante, Valeria Tutino, Francesco Russo, Valentina De Nunzio, Sergio Coletta, Raffaele Armentano, Alberto Crovace, Maria Gabriella Caruso, Antonella Orlando, Maria Notarnicola

**Affiliations:** 1 Laboratory of Nutritional Biochemistry, National Institute of Gastroenterology, “S. de Bellis” Research Hospital, 70013 Castellana Grotte (BA), Italy; isabella.gigante87@gmail.com (I.G.); valeria.tutino@irccsdebellis.it (V.T.); valentinadx@hotmail.it (V.D.N.); 2 Laboratory of Nutritional Pathophysiology, National Institute of Gastroenterology, “S. de Bellis” Research Hospital, 70013 Castellana Grotte (BA), Italy; francesco.russo@irccsdebellis.it (F.R.); antonella.orlando@irccsdebellis.it (A.O.); 3 Histopathology Unit, National Institute of Gastroenterology, “S. de Bellis” Research Hospital, 70013 Castellana Grotte (BA), Italy; sergiofalaut@hotmail.it (S.C.); raffaele.armentano@irccsdebellis.it (R.A.); 4 Animal Facility, National Institute of Gastroenterology, “S. de Bellis” Research Hospital, 70013 Castellana Grotte (BA), Italy; alberto.crovace@irccsdebellis.it; 5 Ambulatory of Clinical Nutrition, National Institute of Gastroenterology, “S. de Bellis” Research Hospital, 70013 Castellana Grotte (BA), Italy; gabriella.caruso@irccsdebellis.it

**Keywords:** cannabinoid receptors, irritable bowel syndrome, ketogenic diet, rat model

## Abstract

The administration of a ketogenic diet (KD) has been considered therapeutic in subjects with irritable bowel syndrome (IBS). This study aimed to investigate the molecular mechanisms by which a low-carbohydrate diet, such as KD, can improve gastrointestinal symptoms and functions in an animal model of IBS by evaluating possible changes in intestinal tissue expression of endocannabinoid receptors. In rats fed a KD, we detected a significant restoration of cell damage to the intestinal crypt base, a histological feature of IBS condition, and upregulation of CB1 and CB2 receptors. The diet also affected glucose metabolism and intestinal membrane permeability, with an overexpression of the glucose transporter GLUT1 and tight junction proteins in treated rats. The present data suggest that CB receptors represent one of the molecular pathways through which the KD works and support possible cannabinoid-mediated protection at the intestinal level in the IBS rats after dietary treatment.

## 1. Introduction

The low-carbohydrate diet based on a higher fat to carbohydrate ratio is called the ketogenic diet (KD). It primarily consists of high amounts of fat, moderate amounts ofproteins, and verylow amounts ofcarbohydrates [1]. Several pieces of evidence have demonstrated beneficial effects of specific diets, including dietary regimens such as low-fermentable, oligo-, di-, monosaccharides and polyols (FODMAPs) and KD in some gastrointestinal (GI) pathological conditions (inflammatory bowel diseases—IBDs) [2] as well as extra-GI diseases (e.g., neurological disorders) [3].

The KD induces a metabolic switch to use fat as a main source of energy, promoting an increase in fatty acids, ketone bodies, and pyruvic acid [4]. Recent studies have demonstrated that KD improves cell oxidative stress and exerts anti-inflammatory effects based on the activation of the peroxisome proliferator-activated receptor-gamma (PPAR-γ) [4,5]. According to the current literature, it can also be speculated that ketone bodies may promote healthy ageing and have effects in alleviating the burden of metabolic disease [6,7], being a useful adjuvant during the treatment of certain cancers [8].

In intestinal inflammatory chronic diseases, the KD administration has been demonstrated to reduce blood glucose levels and affect the gut microbiota, leading to increased beneficial bacteria and reduced proinflammatory microbial populations [9]. Additionally, a personalized dietary approach with low carbohydrates has also been considered therapeutic for irritable bowel syndrome (IBS) subjects since nutrient deficiencies have often been reported in these patients [10].

IBS is a chronic disorder of GI function associated with impaired GI motility, alteration of the brain–intestine axis, and visceral hypersensitivity [11]. A high percentage of these patients have clinical symptoms, mostly abdominal pain and bloating, that often occur immediately after eating certain foods. Milk and dairy products, wheat products, cabbage, onion, peas, beans, hot spices, and fried foods are the main culprits for symptoms [12].

There is an increased interest in using low-carbohydrate diets, such as KD, in IBS treatment [13]. In this framework, a positive effect was demonstrated when using a very low-carbohydrate diet (VLCD) in patients suffering from IBS-D (the diarrhoea variant of IBS) [14]. These patients experienced a better symptom profile during the diet. Nevertheless, studies are few and mainly performed on a small cohort of patients over a short time, and there are currently no other studies showing a long term benefit on the GI tract.

The endocannabinoid (EC) system is closely involved in different GI functions, such as motility, secretion, visceral hypersensitivity, and inflammation [15]. Two of the leading molecular players of the EC system are the cannabinoid receptors, CB1 and CB2; CB1 receptors (CB1Rs) are mainly expressed in the central nervous system and some peripheral tissues, including heart, lung, prostate, ovary, and the GI tract, whereas CB2 receptors (CBR2s) are present almost exclusively in peripheral tissues [16]. Cannabinoid receptors play a central role in GI motility and mediating the pharmacological effects of cannabinoids; hence, Storr et al. have indicated the EC system as a potential therapeutic targeting for IBS management [17]. Other studies have demonstrated that CB1 receptor protein expression could be influenced by the diet, particularly by KD [18,19].

Additionally, the EC can affect postprandial glycaemia, and Troy-Fioramonti et al. [20] have described that GI motility can be induced by anandamide, an endogenous cannabinoid that binds CB receptors, leading to a concurrent reduction in plasma glucose.

In this context, the glucose transporters (GLUTs) play a pivotal role. GLUTs are a tissue-specific membrane protein family involved in absorbing food-derived monosaccharides in the GI tract [21]. In the small intestine of mammals, GLUT1, GLUT2, and GLUT5 have been detected [22]; GLUT1, also known as solute carrier family 2, determines glucose transport across the plasma membranes of animal cells [23]. An overexpression of GLUT1 has been associated with reduced circulating glucose levels. Moreover, the silencing of the Glut1 gene inhibits proliferation and promotes apoptosis of colorectal cancer cells by inactivating the TGF-β/PI3K-AKT-mTOR signaling pathway [24].

In light of our recent data on the beneficial effects of other dietetic approaches to IBS, including the use of a low-FODMAP diet [25], the purpose of the study was to investigate the molecular mechanisms by which low-carbohydrate diets, such as KD, can improve GI symptoms and functions in an experimental model of IBS. Therefore, we evaluated whether KD could affect the intestinal tissue expression of CB receptors, the main proteins involved in regulating intestinal permeability and glucose transport.

## 2. Results

### 2.1. IBS Animal Model Validation

In the present study, the maternal and neonatal separation model was used [26]. This model has been developed as a psychosocial stressor to reproduce the IBS symptomatology [27]. The stress consists of removing the puppies from the mother for three hours per day during the first two weeks of life. Since maternal care affects the hypothalamic–pituitary–adrenal axis and cognitive and emotional functions, maternal separation causes stable changes in the central nervous system of these animals. At the intestinal level, maternal separation promotes in adult animals the development of a condition characterized by visceral hypersensitivity, a typical sign of IBS.

Table 1 describes the three animal groups studied: a control group of n° 12 rats without maternal separation and fed standard diet (CTR); a group fed a standard diet (IBS-St, n° 11 rats), and a rat group fed a low-carbohydrate, high-fat ketogenic diet (IBS-KD, n° 17 rats).

Rats were checked every day, evaluating different parameters and recording for each of them a score from 0 (absent) to 2 (evident) to calculate the possible onset of pain and suffering. The parameters evaluated regarding the degree of suffering and stress-induced experimentally were blepharospasm, hollow cheeks, abnormal position of the ears and the whiskers, appetite loss, and liquid stools.

None of the above parameters was visible in our rat groups during the study, except for a slowdown in the growth of puppies with maternal separation, which showed lower weights compared to the puppies of the control group.

### 2.2. Histological Findings

**Figure 1:**
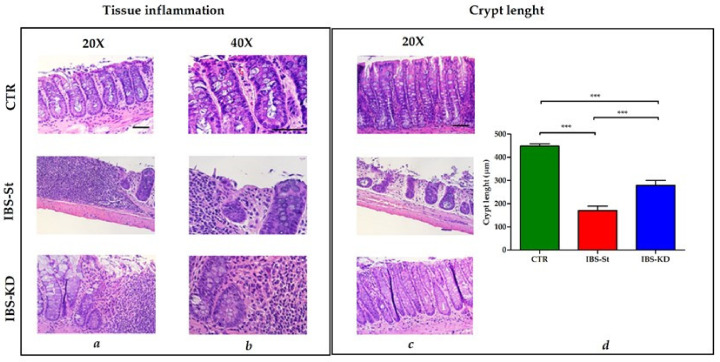
Hematoxylin and eosin staining of distal intestinal tract section from three experimental groups, control group (CTR, n° 12 animals), IBS rats fed a standard diet (IBS-St, n° 11 animals), and IBS rats treated with low-carbohydrate, high-fat ketogenic diet (IBS-KD, n° 17 animals). (**a**) Magnification 20× and (**b**) magnification 40×, scale bars 50 µm. (**c**) Magnification 20×, scale bar 50 µm, shows intestinal crypt length in the control group (CTR), IBS rats fed a standard diet (IBS-St), IBS rats treated with low-carbohydrate, high-fat ketogenic diet (IBS-KD). (**d**) Crypt length in the graph; results represent the mean ± SD (one-way ANOVA with Tukey’s multiple comparison test *** *p* < 0.0001).

The histological analysis was performed upon the sacrifice after 10 weeks of treatment. Each case section of 3 μm-thick, deparaffined and stained with hematoxylin–eosin routine method and histochemical method for mucins (PAS) was analyzed. A score from 0 to 3 was assigned: 0 = absence of inflammation, 1 = mild inflammation, 2 = moderate inflammation, and 3 = severe inflammation. The sections were also considered for plasmacytosis and basal gap/ulceration, length of the crypt and mucin depletion. Figure 1 shows normal intestinal mucosa with intact epithelium in CTR, high inflammatory cell infiltration into mucosa and submucosa in IBS-St, and mild infiltration of mixed inflammatory cells in IBS-KD (Figure 1a,b). The evaluation of intestinal crypt length (Figure 1c) demonstrated no signs of crypt damage in CTR.

In contrast, a gradual loss of the single-cell epithelial layer and cellular damage extending down to the crypt base was visible in IBS rats, irrespective of treatment. A statistically significant difference in crypt length was present between IBS-St and IBS-KD groups (Figure 1d), suggesting that the cellular damage to the crypt base, known to be a peculiar feature of IBS, was partially resolved after KD treatment. The insets of the area zoomed for preparing Figure 1 are shown in Figure S1 in the Supplementary Data.

In a preliminary set of experiments, the study useda group of rats without maternal separation and fed a low-carbohydrate, high-fat KD (CTR-KD). The histological analysis showed minimal changes compared to CTR rats fed a standard diet with only a few inflammatory elements and no different lengths of the intestinal villi and the crypt (Figure S2, Supplementary Data).

### 2.3. Expression of CB Receptors

After ten weeks of KD treatment, we evaluated gene and protein expressions of CB1R and CB2R in intestinal tissue samples from CTR, IBS-St, and IBS-KD groups.

**Figure 2:**
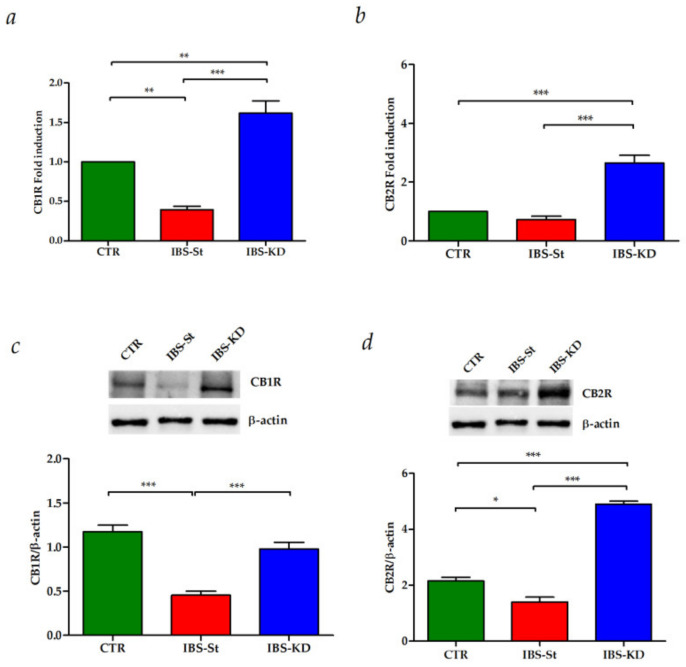
(**a**) CB1 gene expression levels in the control group (CTR), IBS rats fed a standard diet (IBS-St), and IBS rats treated with low-carbohydrate, high-fat ketogenic diet (IBS-KD). (**b**) CB2 gene expression levels in the control group (CTR), IBS rats fed a standard diet (IBS-St), and IBS rats treated with low-carbohydrate, high-fat ketogenic diet (IBS-KD). (**c**) CB1 protein expression levels and representative blots from the control group (CTR), IBS rats fed a standard diet (IBS-St), and IBS rats treated with low-carbohydrate, high-fat ketogenic diet (IBS-KD). (**d**) CB2 protein expression levels and representative blots from the control group (CTR), IBS rats fed a standard diet (IBS-St), and IBS rats treated with low-carbohydrate, high-fat ketogenic diet (IBS-KD). All data represent the results of three different experiments (mean ± SD). *p*-value was determined by ANOVA with Tukey’s multiple comparison test * *p* <0.05, ** *p* < 0.01, and *** *p* < 0.001.

Figure 2 shows the effects of KD on the mRNA levels of CB1R (panel a) and CB2R (panel b) in the three experimental rat groups. A statistically significant decrease in the gene expression levels of CB1R was detected in IBS rats fed a standard diet compared to the control group.

The KD treatment caused a significant overexpression of both CB1R and CB2R genes compared to both the control rats and the standard diet. The same behavior was observed for CB1R and CB2R protein expression (Figure 2c,d, respectively).

**Figure 3:**
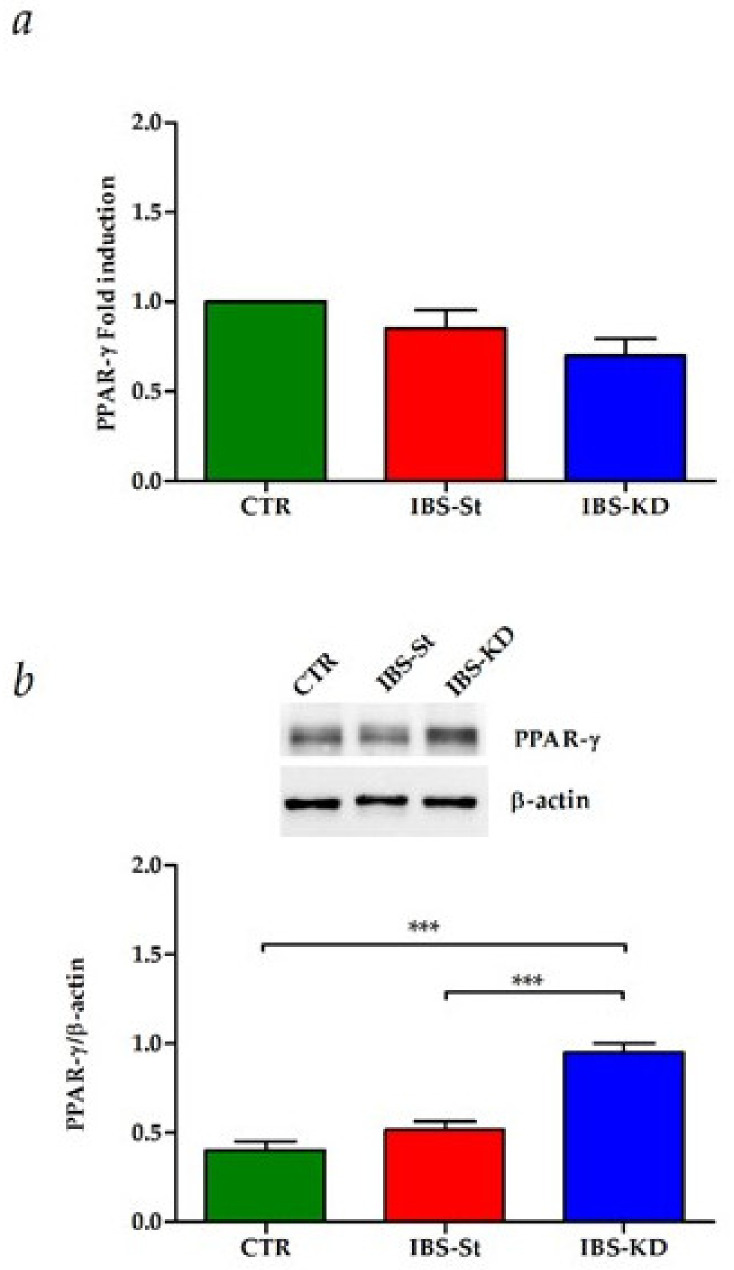
(**a**) Peroxisome proliferator-activated receptor-gamma (PPAR-γ) gene expression levels in the control group (CTR), IBS rats fed a standard diet (IBS-St), and IBS rats treated with low-carbohydrate, high-fat ketogenic diet (IBS-KD). (**b**) PPAR-γ protein expression levels, and representative blots in the control group (CTR), IBS rats fed a standard diet (IBS-St), and IBS rats treated with low-carbohydrate, high-fat ketogenic diet (IBS-KD). All data represent the results of three different experiments (mean ± SD); *p*-value was determined by ANOVA with Tukey’s multiple comparison test; *** *p* < 0.001.

Moreover, in the same samples, no difference was present in the PPAR-γ gene expression among the three groups studied (Figure 3a), whereas KD was able to induce a significant overexpression of PPAR-γ protein, downstream factor tightly linked to function of CB receptors, compared to control and IBS-St (Figure 3b).

### 2.4. GLUT1 Receptor, Tight Junction Protein and Inflammation Marker Evaluation

To evaluate the KD effects on glucose metabolism and intestinal membrane permeability, we studied the gene and protein expressions of the GLUT1 receptor, E-cadherin, Occludin, and Claudin-1.

**Figure 4:**
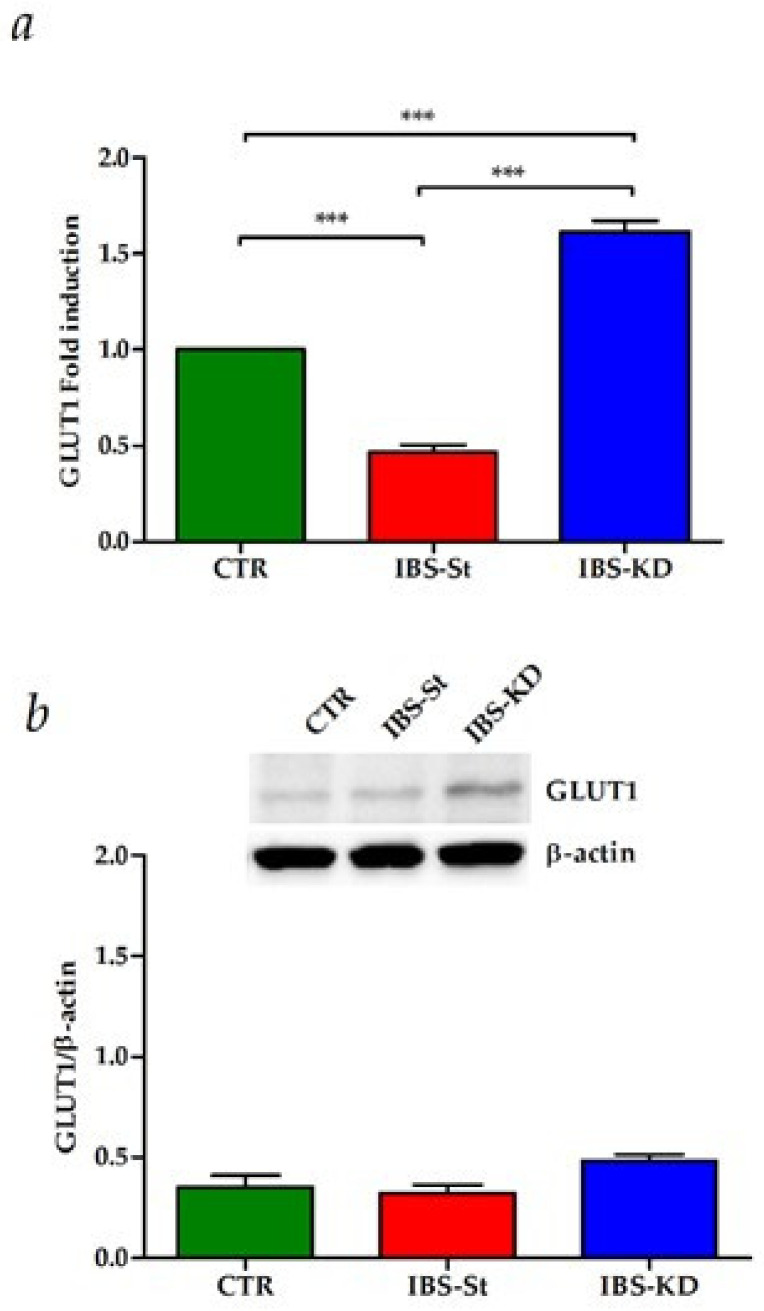
(**a**) GLUT1 gene expression levels in the control group (CTR), IBS rats fed a standard diet (IBS-St), and IBS rats treated with low-carbohydrate, high-fat ketogenic diet (IBS-KD); (**b**) GLUT1 protein expression levels and representative blots in the control group (CTR), IBS rats fed a standard diet (IBS-St), and IBS rats treated with low-carbohydrate, high-fat ketogenic diet (IBS-KD). All data represent the results of three different experiments (mean ± SD). *p*-value was determined by ANOVA with Tukey’s multiple comparison test; *** *p* < 0.001.

The maternal separation induced significantly lower levels of GLUT1 compared to the CTR group. Oppositely, compared to the standard diet, KD was not only able to restore the gene expression levels to those detected in the control group, but it also allowed a significant upregulation of GLUT1 mRNA levels (Figure 4a). In the same group, GLUT1 protein levels were slightly increased compared to controls (Figure 4b).

**Figure 5:**
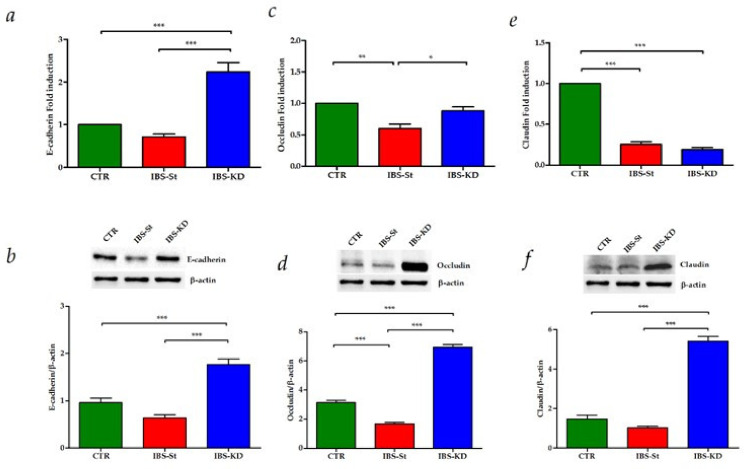
(**a**,**b**) E-cadherin gene and protein expression levels, respectively, detected in the control group (CTR), IBS rats fed a standard diet (IBS-St), and IBS rats treated with low-carbohydrate, high-fat ketogenic diet (IBS-KD). (**c**,**d**) Occludin gene and protein expression levels, respectively, detected in the control group (CTR), IBS rats fed a standard diet (IBS-St), IBS rats treated with low-carbohydrate, high-fat ketogenic diet (IBS-KD). (**e**,**f**) Claudin-1 gene and protein expression levels, respectively, detected in the control group (CTR), IBS rats fed a standard diet (IBS-St), and IBS rats treated with low-carbohydrate, high-fat ketogenic diet (IBS-KD). All data represent the results of three different experiments (mean ± SD). *p*-value was determined by ANOVA with Tukey’s multiple comparison test * *p* < 0.05, ** *p* < 0.01, and *** *p* < 0.001.

The analysis of tight junction (TJ) proteins’ expression showed that the mRNA and protein levels of E-cadherin and Occludin were induced by KD treatment in IBS rats compared to both control and IBS-St (Figure 5a–d respectively). For Claudin-1, no difference in mRNA levels was detected between IBS-St and IBS-KD group, probably due to a rapid mRNA turnover rate (Figure 5e). However, the treatment with KD caused a significant overexpression of Claudin-1 protein (Figure 5f) compared to the other groups.

**Figure 6:**
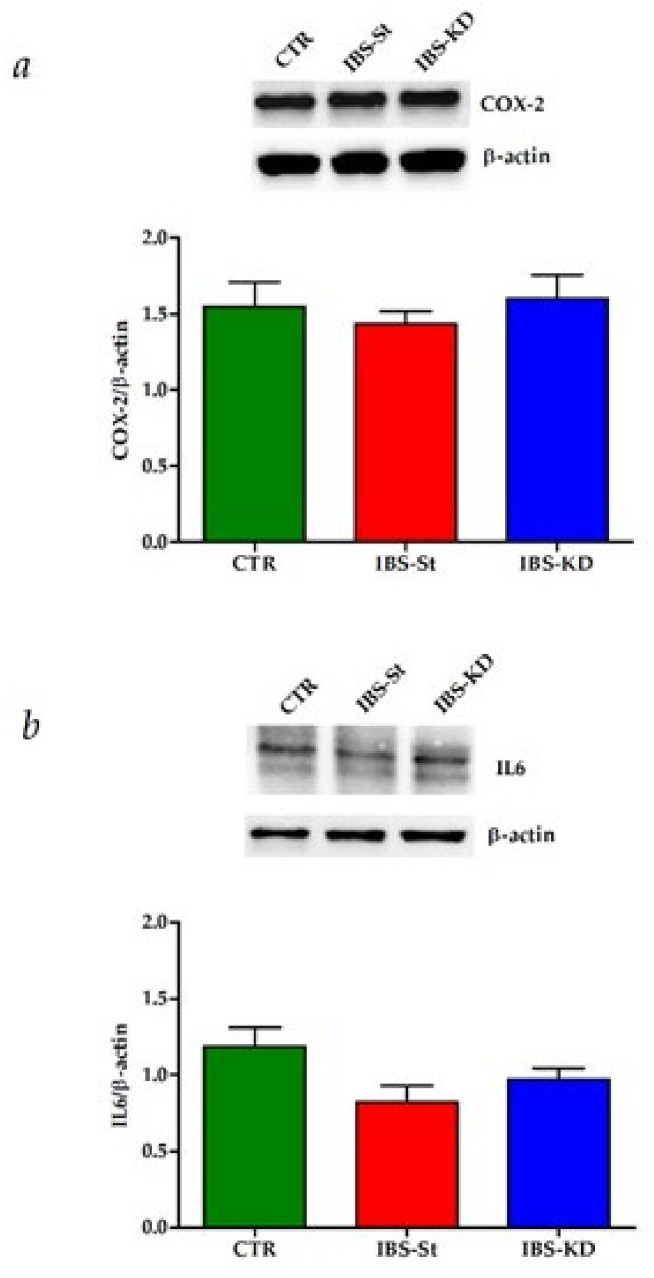
(**a**) COX-2 protein expression levels and representative blots detected in the control group (CTR), IBS rats fed a standard diet (IBS-St), and IBS rats treated with low-carbohydrate, high-fat ketogenic diet (IBS-KD). (**b**) IL-6 protein expression levels and representative blots detected in the control group (CTR), IBS rats fed a standard diet (IBS-St), and IBS rats treated with low-carbohydrate, high-fat ketogenic diet (IBS-KD). All data represent the results of three different experiments (mean ± SD). p-value was determined by ANOVA Tukey’s multiple comparison test.

The intestinal tissue inflammation status in our samples was assessed by evaluating the expressions ofCOX-2 and IL6. No significant difference was observed among the three rat groups regarding the gene and protein expressions of these inflammatory molecular markers (Figure 6a,b).

## 3. Discussion

The present study aimed to investigate how KD can improve GI symptoms and functions in an IBS experimental model by evaluating possible modifications of the expression of CB receptors in the intestinal tissue. Newborn Wistar rats were subjected to stress through maternal separation, an animal model proven to reproduce a condition similar to IBS in an experimental condition [27].

IBS is a minimal inflammatory disorder that affects the gut. The cause of the IBS onset is not precisely known, but it may be multifactorial [28]. A central role in the etiopathogenesis of IBS has been attributed to psychosocial stress, recognized as a factor responsible for the chronic progression of the disease. The possibility of interrupting the brain–gut axis dysfunction through specific dietary treatment could allow novel therapeutic strategies based on their neuroprotective properties [29].

To date, there is no definitive cure for this condition, but many options to relieve symptoms, including lifestyle changes and dietary modifications, are available. Unfortunately, IBS has no specific and unique diet since people react differently to different foods. Usually, many IBS patients find it helpful to increase dietary fiber, drink water, and takeprobiotics. However, in addition to some precautions that each patient can have on diet, some specific nutritional regimens have been proposed, whose intake can positively affect the quality of life of IBS patients. Among them, KD has been considered a potential therapeutic alternative [30].

The effect of a ketogenic microenvironment, induced in exogenous and endogenous manners, is often investigated when shedding light on the understanding of the pathogenesis of various intestinal inflammatory states. One of the effects resulting from very low carbohydrate and KD consumption is the increase incirculating ketone bodies. It is demonstrated that KD alters the human and mouse gut microbiota differently from other diets [2]. Wang et al. have shown that inhibition of the PI3K/Akt/mTOR signaling pathway increases intestinal cell differentiation [31]. Additionally, ketogenesis has a role in intestinal cell differentiation and may inhibit abnormal growth of intestinal cells [32]. As a rule, the impact of KD consists of a shift toward proteolytic fermentation, leading to a reduction in intestinal mucosa inflammation [33].

Moreover, experimental evidence has demonstrated that mitochondria, being the energy-producing organelles of the cell, play a central role in the KD action mechanism [34]. The strategic function of mitochondria in ensuring the KD therapeutic efficiency is due to their role in generating most of the cellular energy [35]. Notably, there are indications that KD may indirectly modulate mitochondrial respiration and energy production [34].

The contribution of the EC system in the IBS pathogenesis is supported by the presence of an altered expression of CB receptors in IBS patients at the intestinal level [36]. Both CB1R and CB2R have a documented protective role in inflammation-associated motility alterations [37]. Treatment with exogenous cannabinoids attenuates inflammation in experimental models of colitis [38], and the use of selective agonists in receptor-deficient mice leads to an attenuation of visceral sensitivity and inflammation [39]. In this context, the use of CB receptor antagonists, such as rimonabant, initially tested as a potential antiobesity drug, has also been shown to modulate GI motility in patients with IBS [40].

In line with the pieces of evidence showing the involvement of the EC system in the regulation of inflammation and GI function, our findings of the upregulation of CB1R and CB2R in rats fed a KD suggest that the manipulation of the EC system signaling pathway can be considered an effective strategy in IBS treatment.

High intestinal tissue levels of mRNA and protein of both CBRs detected after KD treatment demonstrated that CB receptors in the GI tract were functional.

Aside from direct activation of CB receptors, the present data suggest that these receptors represent one of the molecular pathways through which the KD works, evidencing cannabinoid-mediated protection at the intestinal level in the IBS rats after KD treatment. In other inflammation models, cannabinoids have shown anti-inflammatory properties by reducing the chemotaxis of activated T cells [41]. Recently, CB receptor activation on immune cells, as well as colonocytes, has been demonstrated to be crucial in preventing colonic inflammation [42].

Endocannabinoids are molecules released by cell stress, and they can promote both cell survival and cell apoptosis, mostly via the CB1 receptor [43]. The activation of the EC system appears to be associated with energy seeking and storage [44]. Thus, it might act to maintain energy supply, modulating mitochondrial function, resolve inflammation, and repair stress-induced cell damage [43].

The induction of the CB1 receptor and the overexpression of PPAR-γ protein observed in this study support the role of the EC system in modulating inflammation in IBS, a chronic functional GI disorder characterized by a lowgrade of intestinal inflammation [42,45].

CBRs are also known to influence glucose homeostasis [46]. Our present data show that the increase inintestinal CB receptor expression was associated with an upregulation of GLUT1 glucose transporter gene expression. In the intestinal epithelium, the absorption of diet-derived glucose depends on glucose transporters [46], located on the luminal surface and the epithelial cells’ basal side [47]. This study confirms GLUT1 isoform in the rat intestine and suggests the critical role of this receptor in glucose uptake control.

Along with the increase inGLUT1 expression, we observed an overexpression of TJ proteins in our tissue samples after KD treatment. According to our data on the capacity of KD to restore the intestinal crypt length, we observed an improvement of intestinal barrier function, demonstrated from the increase inthe gene expression of E-cadherin, Occludin, and Claudin-1 genes along with their protein levels after dietary treatment.

The intercellular tight junctions (TJs) are classified as single or complex proteins of the mucosal barrier. Alterations in barrier function allow the exposition of luminal factors to the immune cells in the lamina propria, causing the activation of immune processes and inflammation in patients with IBS [48]. Moreover, the length of the intestinal villi and the depth of the crypt are considered valid indicators to evaluate the function of the gut barrier [49].

Several studies claim that barrier restoration improves the symptom profile of IBS patients [50], and experimental studies have shown that the loss of intestinal barrier associated with an increased membrane permeability can cause or affect IBS disease [51]. Therefore, if a specific diet can prevent or restore intestinal barrier dysfunction through the upregulation of TJ proteins, it can be considered a valid therapeutic approach to treat IBS. In this connection, our data also seem to support the feasibility of KD as a proper nutritional regimen in IBS patients.

Although the major flaw of KD is that this diet is too restrictive, potentially determining a decreased microbial diversity with depletion of bifidobacteria [52], its benefits are mainly derived from a constant intake of healthy fats. While high-fat meals can be problematic for these subjects, a moderate intake of healthy fats is possible and beneficial for a short period.

This study has some limitations. The first is the need to investigate the biological significance of biochemical and morphological changes induced by KD, through functional studies in vivo. Additionally, the relatively smallnumber of determinants of intestinal permeability studied does not allow to draw firm conclusions.

However, our findings showing a positive effect on CB receptors involved in regulating intestinal permeability and glucose transport suggest that a definite period of ketosis induced by KD could be beneficial for treating IBS. These data need to be replicated in other experimental studies and, above all, further confirmed in large clinical trials conducted on IBS patients.

## 4. Materials and Methods

### 4.1. Animals and Experimental Design

The study was approved by the Italian Ministry of Health (approval date: November 28, 2018, n. 901/2018-PR) according to European Union guidelines (Directive 2010/63/EU for animal experiments). The animals were housed at the animal facility of the National Institute of Gastroenterology “S. De Bellis” Research Hospital, Castellana Grotte, Bari, Italy. All the applied procedures followed the International Guidelines for the use of laboratory animals, minimizing animal suffering.

The animal model chosen is newborn Wistar rats subjected to stress through maternal separation (MS) to induce IBS in adulthood [27]. Stress was due to removing the puppies from the mother for 3 h a day during the first two weeks of their life.

The experimental design provided that, after weaning, the animals subjected to MS were further divided into two subgroups—one group fed a standard diet (IBS-St, n° 11 rats) and one group fed a low-carbohydrate, high-fat ketogenic diet (IBS-KD, n° 17 rats). A control group of n° 12 animals without maternal separation and fed standard diet was also included (Table 1). Diets were supplied as pellets (4RF21 standard diet and KD purchased by Mucedola Srl, Settimo Milanese, Italy) and administered for ten weeks. After treatment, the animals were sacrificed by anesthetic overdose, and the entire intestinal tract was immediately removed and washed with cold phosphate-buffered saline. The small intestine and colon were cut along the mesenteric insertion, placed on a paper strip at 0–4 °C and analyzed through a stereomicroscope at 3× magnification. A part of the distal small intestine was immediately put into liquid nitrogen for real-time Polymerase Chain Reaction (PCR) and Western blotting analyses, and the remaining part was fixed in 10% neutral buffered formalin for 24 h and embedded in paraffin in a “Swiss roll” fashion.

### 4.2. Histology

Tissue sections from the small intestine were fixed in 10% buffered formalin, dehydrated and paraffin-embedded. Three-micrometer-thick sections from the proximal, medial, and distal intestinal tracts were stained using a hematoxylin and eosin standard protocol. PAS staining on distal small intestine sections was performed to identify mucins. Observations and imaging were performed with a Nikon Eclipse Ti2 (Nikon Inc., Melville, NY, USA)

### 4.3. Gene Expression Assay

RNA was extracted from distal intestine tissue using the Qiagen RNeasy Mini Kit (Qiagen, Hilden, Germany), according to the manufacturer’s instructions. Total RNA concentration was quantified with a Nanodrop ND-1000 spectrophotometer (Thermo Fisher Scientific, Waltham, MA) and purity estimated by 260/280 nm absorption. We reverse-transcribed 2 µg of RNA from each sample using the iScript Reverse Transcription Kit (Bio-Rad). The total cDNA was analyzed using real-time Polymerase Chain Reaction (PCR) for evaluation of CB1R, CB2R, PPAR-γ, GLUT1, E-cadherin, Occludin, Claudin-1, and β-actin on a CFX96 Touch Real-Time PCR Detection System (Bio-Rad Laboratories, Hercules, CA, USA) according to the manufacturer’s instructions. Table 2 shows the gene-specific primer sets used (Bio-Rad Laboratories). The β-actin gene was chosen as the reference gene, and the DDCt method was used for relative quantification by CFX Manager software 2.1 (Bio-Rad Laboratories).

### 4.4. Western Blotting

Distal small intestine samples from control and treated rats were lysed with a buffer (Pierce Ripa buffer, Thermo Scientific, Rockford, IL, USA) supplemented with protease and phosphatase inhibitors to obtain protein extracts (Thermo Scientific, Rockford, IL, USA). The tissue samples were homogenized and centrifuged at 14,000 rpm for 15 min at 4 °C, and protein concentration was measured by a standard Bradford assay (Bio-Rad, Milan, Italy). Aliquots of 50 µg of total protein extract from each sample were denatured in 4× Laemmli sample buffer with 10% β-mercaptoethanol and loaded into 4–12% precast polyacrylamide gels (Bio-Rad, Milan; Italy) for Western blot analysis. After the blotting onto a PVDF membrane (Bio-Rad Laboratories, Milan, Italy), the proteins were probed with the following primary antibodies: CB1R (Abcam, Cambridge, UK), CB2R (Abcam, Cambridge, UK), PPAR-γ (Abcam, Cambridge, UK), GLUT1 (Cell Signaling Technology, Beverly, MA, USA), E-cadherin (Santa Cruz Biotechnology, Santa Cruz, CA, USA), Occludin (Santa Cruz Biotechnology, Santa Cruz, CA, USA), Claudin (Cell Signaling Technology, Beverly, MA, USA), COX-2 (Cell Signaling Technology, Beverly, MA, USA), IL6 (Immunological Science) and β-actin (Cell Signaling Technology, Beverly, MA, USA). After overnight incubation, the membranes were further incubated with a horseradish peroxidase-conjugated rabbit or mouse secondary antibody (Bio-Rad, Milan, Italy). The proteins were detected by chemiluminescence (ECL, Thermo Scientific, Rockford, IL, USA), and the densitometric analysis of each protein-related signal was obtained using the Molecular Imager Chemidoc^TM^ (Bio-Rad, Milan, Italy) and normalized against β-actin expression.

## Figures and Tables

**Table 1:** Experimental groups: no irritable bowel syndrome (IBS) rats fed a standard diet (control group, CTR); IBS rats fed a standard diet (IBS-St); IBS rats treated with low-carbohydrate, high-fat ketogenic diet (IBS-KD).

Experimental Groups	Rats (Number)	Maternal Separation	Treatment
CTR	12	No	Standard diet
IBS-St	11	Yes	Standard diet
IBS-KD	17	Yes	Ketogenic diet

**Table 2:** Primers for quantitative real-time Polymerase Chain Reaction (PCR).

Target Genes	Gene Symbol	ID Assay
Cannabinoid receptor 1 (CB1R)	Cnr1	qRnoCED0008430
Cannabinoid receptor 2 (CB2R)	Cnr2	qRnoCED0008595
Peroxisome proliferator-activated receptor gamma (PPAR-γ)	Pparg	qRnoCID0006036
Solute carrier family 2, facilitated glucose transporter member 1 (GLUT1)	Slc2a1	qRnoCED0003212
Cadherin-1,E-Cad/CTF1 E-Cad/CTF2 E-Cad/CTF3 (E-Cadherin)	Cdh1	qRnoCID0003281
Occludin	Ocln	qRnoCID0005733
Claudin-1	Cldn1	qRnoCED0051349
Actin, cytoplasmic 1 Actin, cytoplasmic 1, N-terminally processed (β-actin)	ACTB	qRnoCID0056984

## Data Availability

Data are available from corresponding author upon reasonable request.

## Supplementary Materials

The following are available online at https://www.mdpi.com/1422-0067/22/6/2880/s1, Figure S1: Histological analysis of four experimental groups in the study (Magnification 10x), Figure S2: Histological analysis in rats group without IBS treated with standard diet (CTR-St) and ketogenic diet (CTR-KD).
