# Supplementary figures and images for: Cannabinoid Receptors Overexpression in a Rat Model of Irritable Bowel Syndrome (IBS) after Treatment with a Ketogenic Diet
Source: Int J Mol Sci. 2021 Mar 12;22(6):2880. doi: 10.3390/ijms22062880 (PMC7999285; doi:10.3390/ijms22062880)

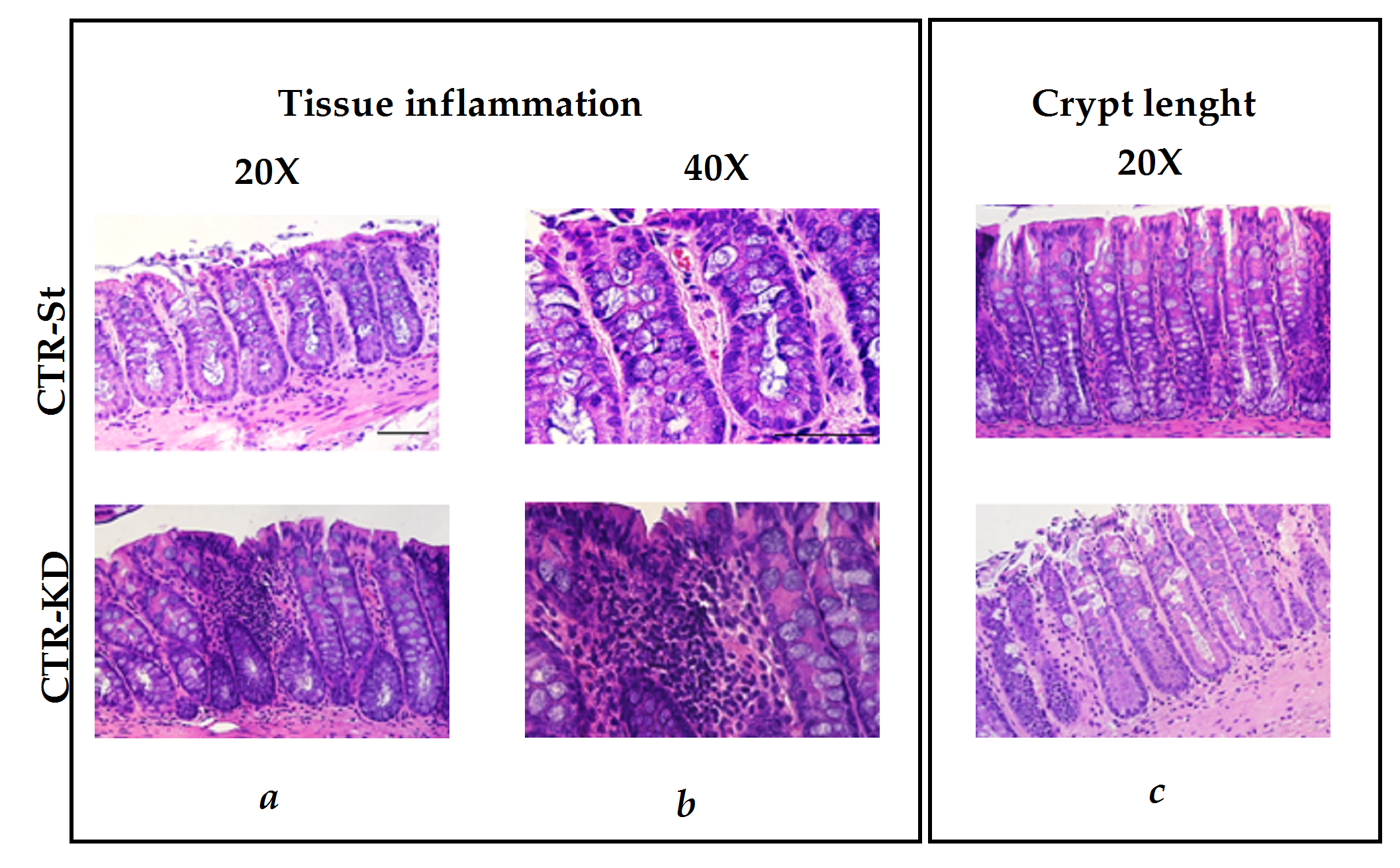

Supplement: Supplementary file 1 [file ijms-22-02880-s001.zip › Figure 2S.tif]

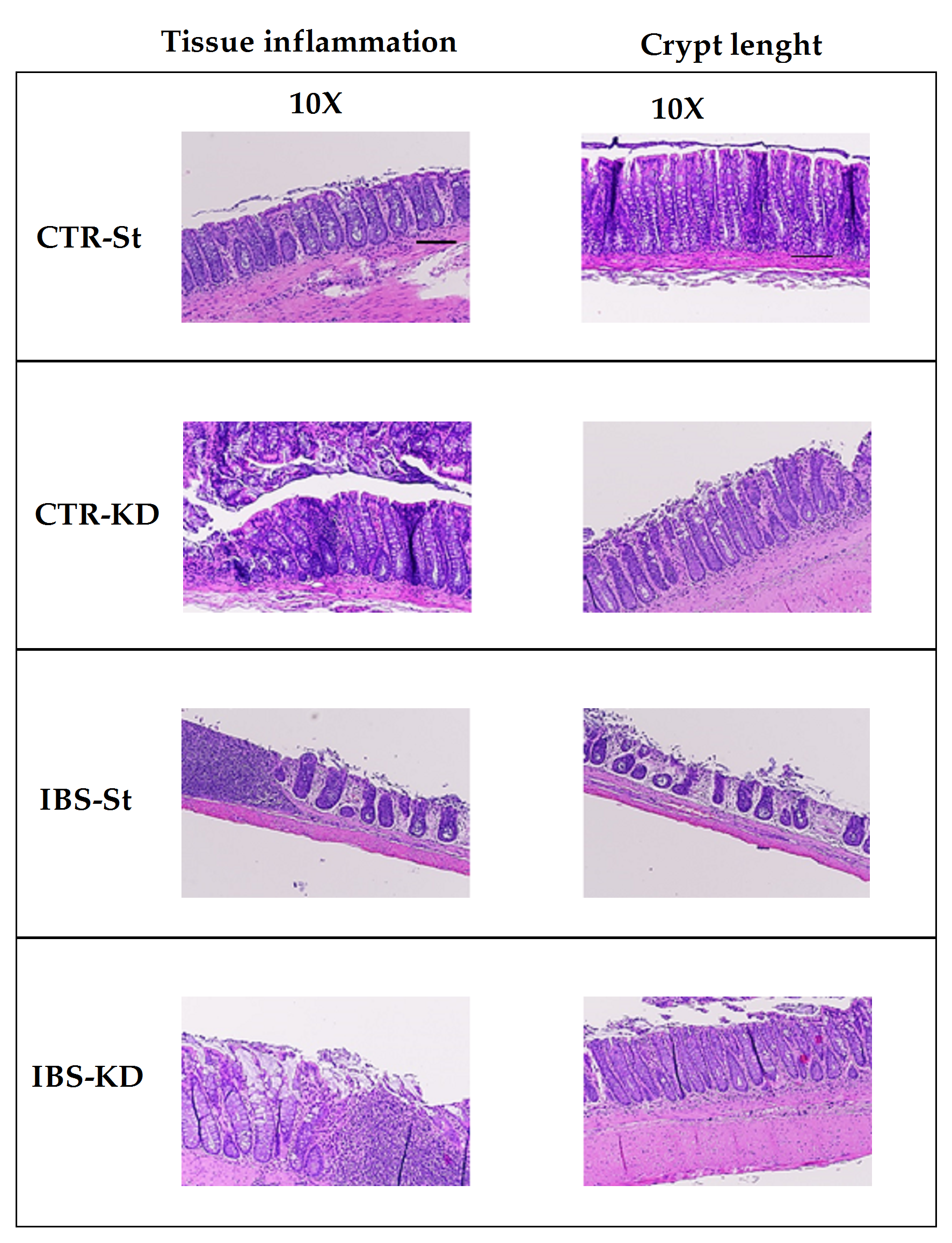

Supplement: Supplementary file 1 [file ijms-22-02880-s001.zip › Figure 1S.tif]
